# An earphone fit deviation analysis algorithm

**DOI:** 10.1038/s41598-023-27794-y

**Published:** 2023-01-19

**Authors:** Yan Yan, Yonghong Liu, Haining Wang

**Affiliations:** School of Design, Hunan University, Changsha, 410082 China

**Keywords:** Psychology, Engineering

## Abstract

This study provides an accurate method for evaluating the fit of earphones, which could be used for establishing a linkage between interference/gap values with human perception. Seven commercial CAD software tools stood out and were explored for the analysis of the deviation between earphone and ear. However, the current deviation analysis method remains to be improved for earphone fit evaluation due to excessive points in the calculation (Geomagic Wrap and Siemens NX), lack of value on interference (Geomagic Control X), computation boundary required (Rapidform XOR/Redesign), repetitive computation with same points and inclined calculation line segment or even invalid calculation (Solidworks, Creo). Therefore, an accurate deviation analysis algorithm was promoted, which calculated the deviation between earphone and ear exactly and classified the interference set and gap set precisely. There are five main procedures of this algorithm, which are point cloud model pre-processing, the generation of distance vectors, the discrimination of interference set and gap set, the discrimination of validity, and statistical analysis and visualization. Furthermore, the usability and validity of the deviation analysis algorithm were verified through statistical analysis and comparing visual effects based on the earphone-wearing experiment. It is certified that the deviation analysis algorithm is appropriate for earphone fit evaluation and the eight indexes of this algorithm were proved to be related to subjective comfort scores. It is meaningful for ear-worn product fit analysis, design, and development phases.

## Introduction

There is a large market of ear-related products including earmuffs, earplugs, earphones, and hearing aids^1^. The global earphone and headphone market size was valued at USD 34.8 billion in 2020 and is expected to grow at a compound annual growth rate of 20.3% from 2020 to 2027. The ergonomics design and accommodation research of ear-related products is under development. It points out that there is still a conflict between anthropometry and current product dimensions, and the relationship between 3D ear anthropometry and product design has not been sufficiently studied to provide a comprehensive generalization of its application in ergonomic design^2^. Even so, the product design based on those 3D shape characteristics of the human body has resulted in gaining a better fit and increased comfort, satisfaction, safety, and usability^3^.

Ears consist of cartilage covered with skin and arranged in a pattern of various elevations and depressions^4^. The shape of the external acoustic meatus is not a straight canal but passes upward from the external opening. The external ear consists of the auricle and the external acoustic meatus. The auricle collects sound waves and conducts them along the external acoustic meatus inwards towards the eardrum, the tympanic membrane. The component of the auricle includes skin, cartilaginous framework, ligaments, auricular muscles, and bone. In addition, the external acoustic meatus extends from the concha to the tympanic membrane; it is approximately 2.5 cm from the floor of the concha and approximately 4 cm from the tragus. It has two structurally different parts: its lateral third is cartilaginous, and its medial two-thirds is osseous^4^. The biological structure of the ear is so complex that it is difficult to provide a comprehensive application of 3D ear anthropometry in the ergonomic design of earphones^2^. Thus, the relationship between ear anthropometric data and related product design is still required a comprehensive exploration^2^. For improving the user experience of ear-related products, physical fit and comfort perception both need to be taken into consideration. Algorithms are used as specifications for performing calculations and data processing and there are several algorithms that are related to computer vision^5–8^. However, there is no such tool/algorithm that could assist in exploring the earphone fit accurately, which impede the fit design of earphone based on the ear anatomy.

In general, proper fit is typically defined as a small and uniform distance between the shape of the body and products^9^. Generally, three methods, including design for collective fit, design for fit within clusters, and design for an individual fit, are viable in improving the fit of products based on 1D/2D anthropometric data or 3D models^10^. The first strategy is designed for collective fit, or design for average. It mainly links 1D anthropometrical data to product characteristics. In the research of Lee et al.^11^, the earphone-head was designed to refer to the average of concha measurements. The second strategy is to design for fit within clusters. The categorization of measurements is an effective method to associate body shapes with different types of products^12–14^, including principal component analysis^15^, clustering analysis^16–19^, and so on. These methods were used for generating the sizing system of products^15–22^. The third strategy is to design for individual fit. It is widely used for the design of hearing-aid equipment^23^ and customized earphones. Customized earphones, such as UE custom in-ear monitors and QDC in-ear earphones, were conducted to fit the unique shape of ears to provide incredible comfort and noise cancellation.

Apparently, it is better to connect accurate 3D models of human shapes with the entire product surface geometry for assessing proper fit. However, it is illustrated that 3D models were cumbersome to work with for product designers as they are shown in an unmeasurable and highly dimensional abstract space^10^. For presenting advanced information in shape, virtual fit analysis and finite element analysis were frequently used. In virtual fit analysis, the gap means that there is a deviation between the two models. And the inference means that there is an overlap between the two models. In the research of Ellena^9^, virtual fit analysis method was used for investigating and comparing the fitting accuracy of helmets. The standoff distances (SOD) and Gap Uniformity (GU) between the head and helmets were recorded and the helmets fit index was established. For the earphone design, an ear-tip was designed based on a virtual fit analysis of an earphone with 3D ears^11^. The fit of an oxygen mask is assessed by the virtual fit analysis method^20,24^. In this research, Euclidean distances between the mask and facial surface were calculated and design details of shape were adjusted^25^. In addition, finite element analysis (FEA) was used for indicating the distribution of pressure and displacements. In the research of Lee^3^, template registration and FEA were used for predicting the contact pressure between the face and the product. Thus, virtual fit analysis method is a more appropriate way to estimate the fit of wearable products instead of FEA.

## Related work

For virtual fit analysis done previously, the deviation analysis tool in CATIA V5R21™ (Dassault Systemes, Velizy, FR) was frequently applied^9,11^ in ergonomics design. Also, it is widely applied in product design optimisation^26^, additive manufacturing (AM) artifacts, and accuracy comparison^27^.

In industry, deviation analysis is a routine form of troubleshooting performed at process manufacturing facilities. When speed is imperative, a robust deviation detection system, along with a good process for analyzing the resulting data, is essential for solving problems quickly. In dataPARC, the four deviation detection methods were frequently used, including absolute change (the simplest form of deviation detection. Comparing a value against the average), variability (how much the data varies or how to spread out the data is relative to the average), standard deviation (a standard for control charts. Measures how much the data varies over time), multi-parameter (advanced deviation detection metric showing the difference between expected values and real data, to evaluate the overall health of the process. The ranges are often rate-dependent). And in Creo Parametric 8.0, Two types of deviation analysis can be performed, including distance to the surface and geometric. In distance to surfaces mode, it calculates the distance from points in the scan set best-fit surfaces to the corresponding reference surfaces in the design model. And in Geometric mode, it calculates the size (where applicable) of the scan set best-fit surfaces and also the location and orientation of those surfaces relative to their reference surfaces in the design mode. Moreover, the deviation analysis method of CATIA is defined as a diagnostic tool that can calculate the angle between faces. A single edge or a series of edges could be selected. The modality of the edge is diverse, which can be between faces on a surface or any edges on a solid. In addition, the projection direction of the deviation analysis method of CATIA could be defined, by picking a plane or a line.

**Figure 1:**
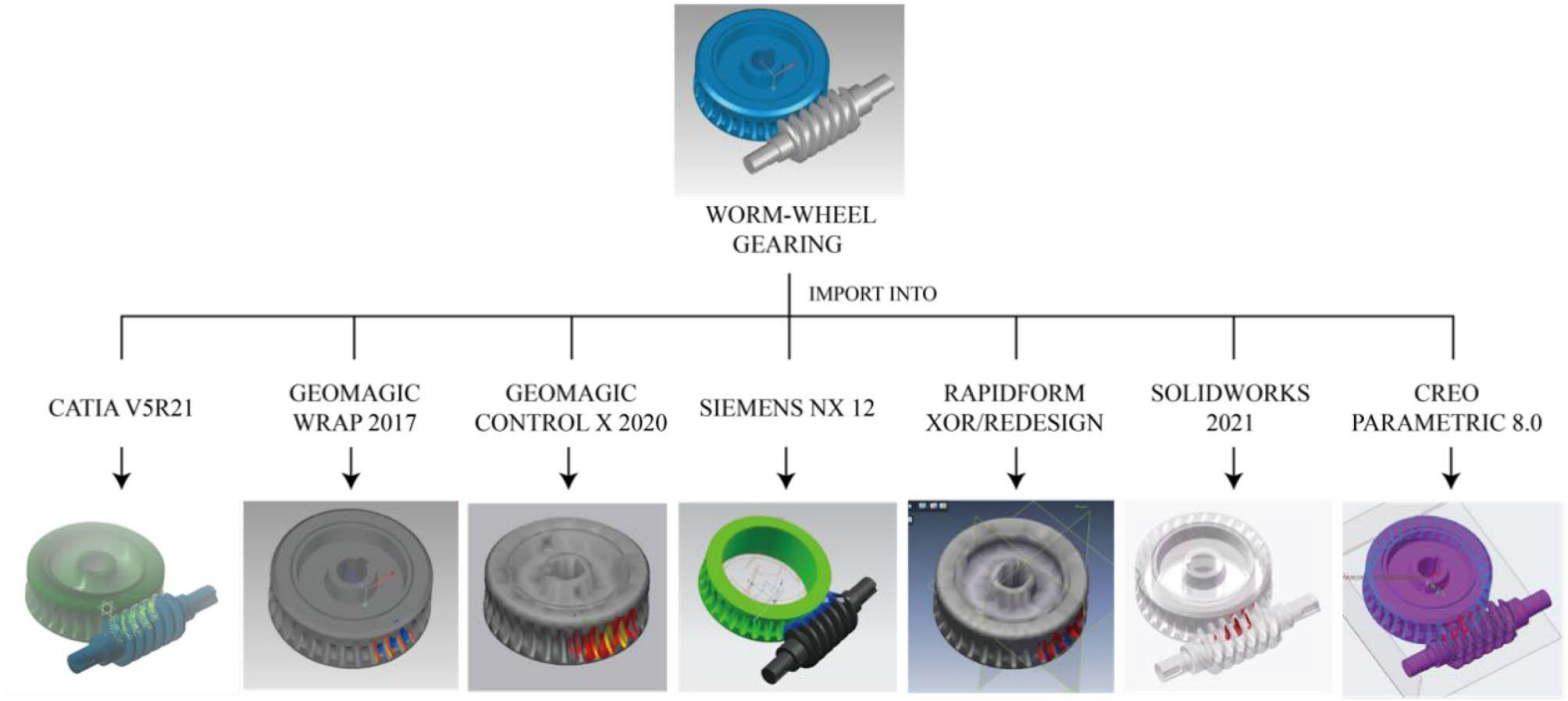
Deviation analysis effect between worm-wheel gearing calculated by seven commercial CAD tools (CATIAV5R21, Geomagic Wrap 2017, Geomagic Control X 2020, Siemens NX 12, Rapidform XOR/Redesign, Solidworks 2019, Creo Parametric 8.0).

According to the Desk research on the deviation analysis method, seven commercial CAD software tools were approved to operate the deviation analysis functions perfectly. They are Geomagic Wrap 2017™ (3D Systems, Rock, Hill, SC,US), Geomagic Control X 2020™ (3D Systems, Rock, Hill, SC,US), Siemens NX 12 (Siemens, Gernamy), Rapidform XOR/Redesign (INUS technology, Korea), Solidworks 2019 (Dassault systems, Paris), and Creo Parametric 8.0(PTC, Las Vegas). In the very first attempt, worm-wheel gearing was imported into these commercial CAD software tools. It turns out that deviation analysis can be conducted in all of them (Fig. 1).

In a manufacturing scenario, deviation analysis is a vital method for reverse engineering, which is used for verifying the deviation between the scanned or modeling models with the reference surfaces. However, only some ergonomic research used the deviation analysis for virtual fit analysis (Table 1). In the research of Meunier^28^, the laser scanner and software ‘shapeanalysis’ were developed to calculate standoff distance, and it proved to be an excellent tool for the assessment of helmet fit. Wonsup lee^24,29^ identified the fit as the infiltration distance between the oxygen mask CAD and 3D face image. When the infiltration distance is more than 10 mm, it means excessive pressure and deep infiltration. And when the infiltration distance is less than 0 mm, it means oxygen leakage. Thierry Ellena^9^ used the Gap Uniformity (GU) and Standoff Distance (SOD) as the independent variables of helmet fit index (HFI), and it provides a fit score from 0 (excessively poor fit) to 100 (perfect fit) was compared with subjective fit assessments of surveyed cyclists, and results showed that HFI was related with participants’ feelings when comparing three commercially available bicycle helmets. Wonsup lee^11^ only mentioned that the ear-tip design could be conducted based on a virtual fit analysis method. However, the detailed evaluating procedures and results were not shown in the papers. According to the listed research, the gap/interference distance could be regarded as an indicator of fit and comfort. In some research, the gape/interference distance was calculated by commercial software with deviation analysis function. Others measured the distance between the wearable product and the human body through CAD software manually. However, none of the previous research mentioned that the appropriate commercial software for earphone fit analysis.

**Table 1:** Ergonomic researches which used the deviation analysis.

Author	Objects	Indexes	Software	Conclusion
Pierre Meunier	Helmet fit	Standoff distance	Shapeanalysis	Three-dimensional laser head scanning offers unparalleled richness of data and analytical power in the calculation of standoff distance
Wonsup Lee	Oxygen mask design	Infiltration distance	Rapidform 2006	Fit was defined as the infiltration distance between the oxygen mask CAD and 3D face image. Deep infiltration ( e.g. infiltration distance > 10 mm) means an excessive pressure, while no infiltration (infiltration distance < 0 mm) can be explained as an oxygen leakage
Thierry Ellnea	Helmet fit	Gap uniformity (GU), Standoff distance (SOD)	CATIA	This paper presents a novel method to investigate and compare fitting accuracy of helmets based on 3D anthropometry, reverse engineering techniques and computational analysis
Wonsup Lee	Ear-tip design	–	Artec studio	The ear-tip can be designed based on a virtual fit analysis of earphone with the 3D ears

## Pilot study

For evaluating the fit between earphones and ears, the deviation analysis functions should be tried as the first attempt. As the proper fit is typically defined as a small and uniform distance between the shape of products and the human body, the variables which can represent the amount and uniformity should be contained in the fit analysis. In addition, the indexes of deviation analysis represent the spatial distance between the two models. Only specific points of the two models should be engaged in fit analysis instead of all points of both models. Therefore, commercial software with deviation analysis functions was applied for evaluating the fit of the earphone at first.

**Figure 2:**
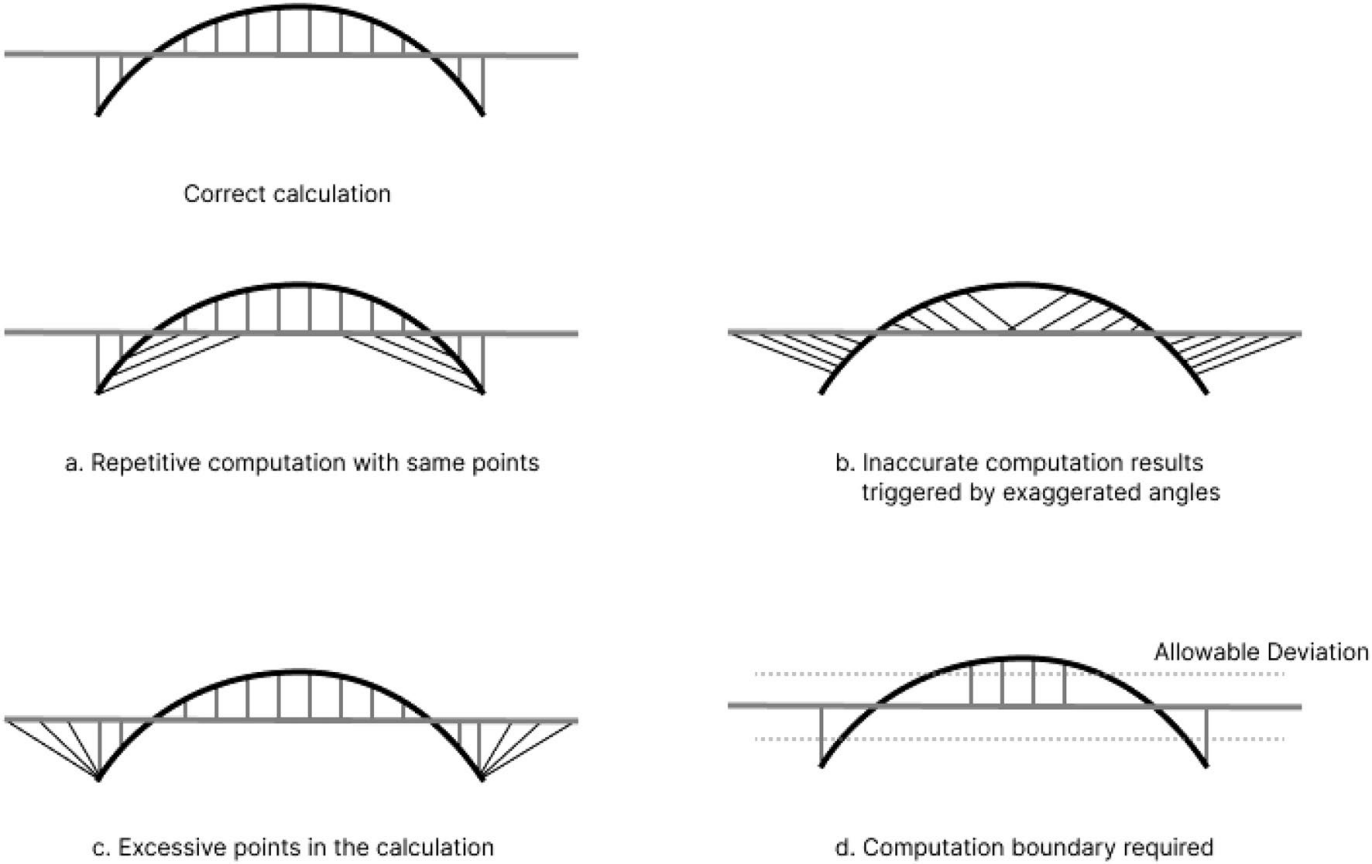
2D diagram of Incorrect calculation situation ((**a**) Repetitive computation with same points; (**b**) Inaccurate computation results triggered by exaggerated angles; (**c**) Excessive points in the calculation; (**d**) Computation boundary required).

For comparison, the same earphone (with only the body portion) and ear (with only the concha part) models were imported into the seven software tools. It found out that deviation analysis between ear and earphones can be conducted on five of them, which are CATIA, Geomagic Wrap, Geomagic Control X, Siemens NX, and Rapidform XOR/Redesign, whereas all of them perform poorly. There are four main incorrect calculation situations for deviation analysis in earphone fit evaluation (Fig. 2).

**Figure 3:**
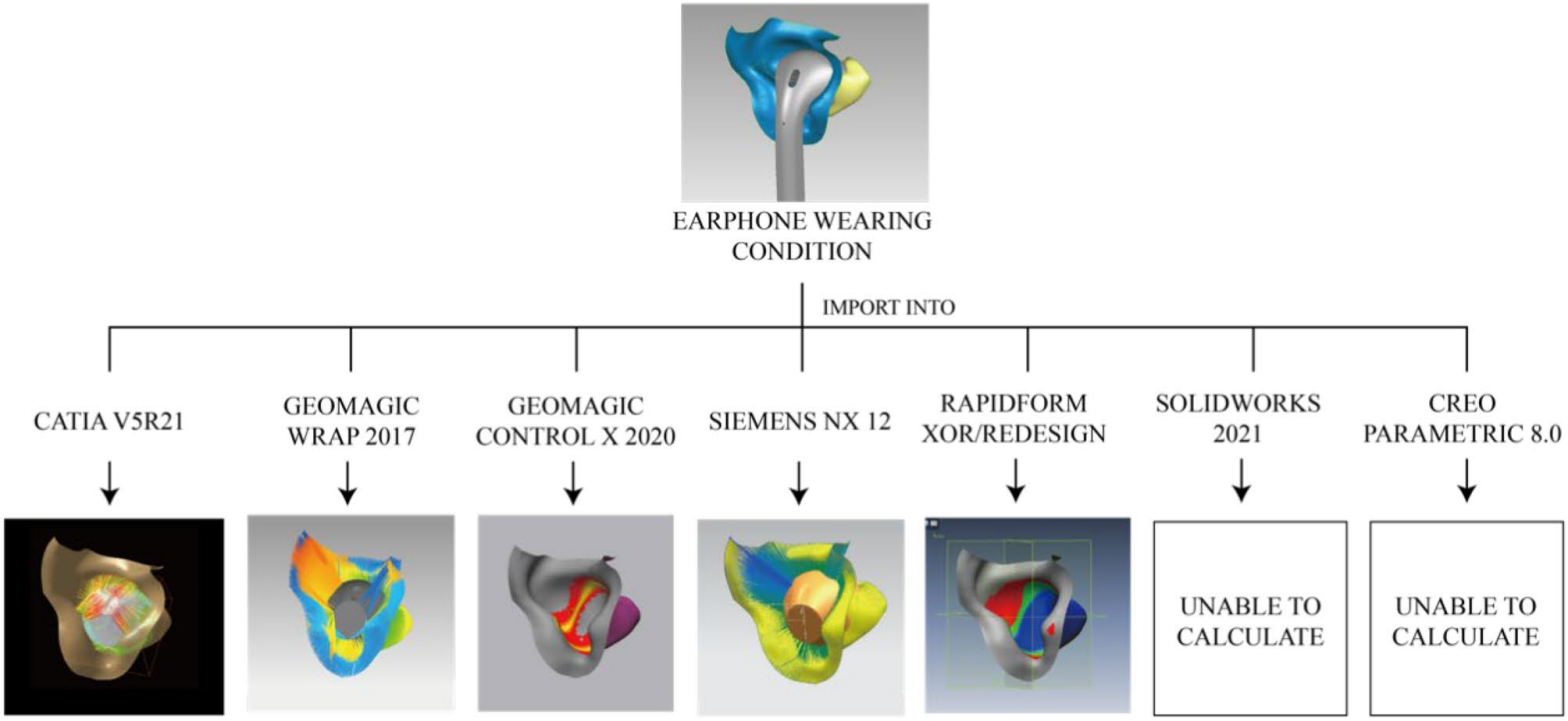
Deviation analysis effect between earphone (with only body portion) and ear (with only concha part) calculated by seven commercial CAD tools (CATIAV5R21, Geomagic Wrap 2017, Geomagic Control X 2020, Siemens NX 12, Rapidform XOR/Redesign, Solidworks 2019, Creo Parametric 8.0).

For CATIA, the ‘deviation analysis’ function was selected and set in ‘only orthogonal’ mode. Six values of deviation analysis appeared. However, there are some mistakes, which are induced by repetitive computation with the same points (Fig. 2a) and inaccurate computation results triggered by exaggerated angles between ear and earphone meshes (Fig. 2b). For Geomagic Wrap, every point of the ear was calculated. However, some of them do not represent the spatial relationship between two meshes. Therefore, excessive points were calculated (Fig. 2c). This incorrect situation is also shown in Siemens NX. In addition, only the maximum gap and maximum interference were shown in the ‘deviation measurement’ method in Siemens NX. Moreover, for Geomagic Control X, only the maximum distance of positive value was counted which is apparently a lack of value of interference. Besides, ‘Allowable Deviation’ should be set before doing ‘Mesh Deviation’ in Rapidform XOR/Redesign (Fig. 2d), while this index is not clear between ear mesh and earphone mesh. In addition, gap and interference cannot be calculated in Solidworks and Creo as both ear mesh and earphone mesh are not assembly parts, and there is not a clear assembly relationship between them. The comparison of functions and values between seven commercial software is shown in Table 2. Moreover, calculation of visual effects between ear mesh and earphone mesh on five commercial CAD software tools were shown in Fig. 3. All in all, the effect of the deviation analysis tool in CATIA is better than others among existing deviation analysis methods in all commercial CAD software.

**Table 2:** Comparison between deviation analysis result of seven commercial CAD software tools on concha and body portion of earphone.

Software	Function name	Distance computation method	Quantifiable index	Value between external ear and earbuds (mm)	Shortage
CATIA V5	Deviation analysis	Euclidean distance	1. Maximum value of interference	− 2.586	1. Repetitive computation with same points 2. Inaccurate computation induced by an exaggerated angle between distance segment and meshes
			2. Mean value of interference	− 0.900	
			3. Maximum value of gap	6.984	
			4. Mean value of gap	2.273	
			5. Mean value of all	1.130	
			6.Standard deviation of all	2.214	
Geomagic Wrap 2017	Deviation analysis	Euclidean distance	1.maximum distance of positive value	− 22.002	All points of ear mesh were calculated
			2. maximum distance of negative value	− 5.925	
			3.mean distance of positive value	18.694	
			4. mean distance of negative value	3.907	
			5.Standard deviation	− 0.370	
			6.RMS	6.456	
Geomagic Control X 2020.1.1	Surface deviation	Euclidean distance	Maximum distance of positive value	2.1165	Only gap was calculated
Siemens NX 12	Deviation measurement	Euclidean distance	Maximum positive tolerance	2.590	All points of ear mesh were calculated
			Maximum negative tolerance	− 16.032	
Rapidform XOR/Redesign	Mesh Deviation	Euclidean distance	–	–	“Allowable Deviation” should be set in advance
Solidworks	Interference Detection	Inference volume	Inference volume	–	Unable to calculate
Creo Parametric 8.0	Global Interference	Inference volume	Inference volume	–	Unable to calculate

The deviation analysis tool in CATIA is not an excellent method for quantifying the virtual fit between the earphone (with only the body portion) and the ear (with only the concha part). Hence, this research aims to promote a more accurate deviation analysis algorithm for investigating the relationship between physical fit and comfort perception of earphones in this research. It is committed to stating the physical fit between ear and earphones precisely and providing physical guidance for holistic, user-centered design for ear-worn products.

## Deviation analysis algorithm for earphone fit analysis

**Figure 4:**
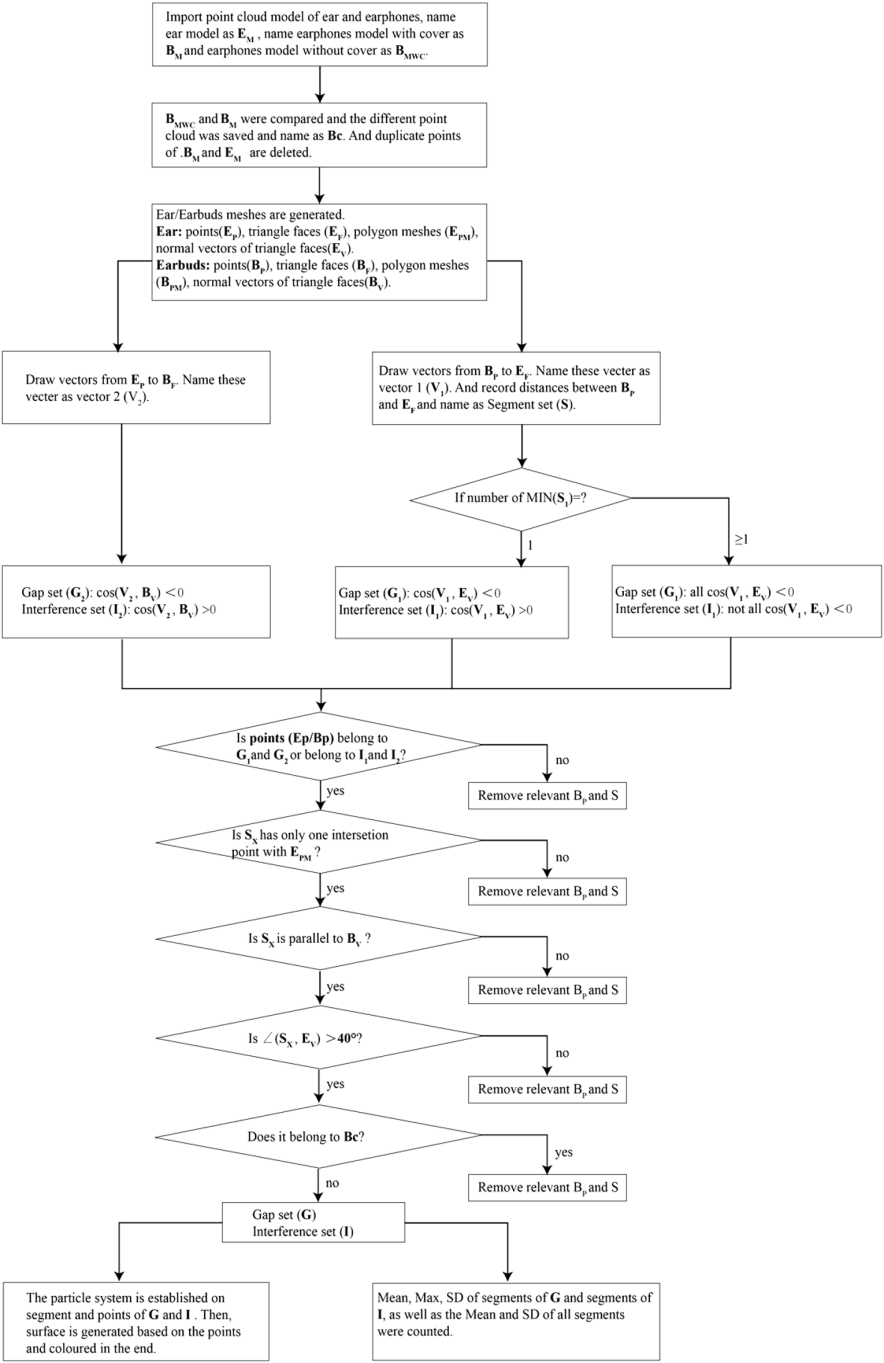
Flow chart of deviation analysis algorithm.

In this program, the ear model is regarded as a reference model and the earphone model is regarded as a target model. Both ear and earphone models are processed as triangular mesh models in STL format. In this algorithm, the gap means that there is a deviation between the two models. And the inference means that there is an overlap between the two models. Thus, the gap set which consists of segments means that there is a gap between the two models, and the interference set which consists of segments means that there is an interference between two models. Briefly, the computational logic of the deviation analysis algorithm is shown in Fig. 4. There are five main procedures of this deviation analysis algorithm in general:

### Point cloud model pre-processing

The ear model ($${E}_{M})$$ is set as the reference model, while the earphone model ($${B}_{M})$$ is set as the target model. The target mesh engaged in calculation is named as $${B}_{MWC}$$. Therefore, the differential part of the target model ($${B}_{C})$$ is:

$$B_{C} = B_{M} - B_{MWC}$$

The reference model includes points (${EP}_{g}$), triangular facets (${EF}_{h}$), polygon $(EPM$), and vertical vectors of triangular facets (${EV}_{h}$). In addition, the target model includes points (${BP}_{g}$), triangular facets (${BF}_{h}$), polygon $(BPM$), and vertical vectors of triangular facets (${BV}_{h}$). a,c represents the number of points of the reference model and target model separately, while, b,d represents the number of surfaces of the reference model and target model separately.

The assemble of the reference model is:

$$({EP}_{g},{EF}_{h},EPM,{EV}_{h})\in {E}_{M}$$

$$(g=1\dots a, h=1\dots b)$$

The assemble of the target model is:

$$({BP}_{i},{BF}_{j},BPM,{BV}_{j})\in {B}_{M}$$

$$(i=1\dots c, j=1\dots d)$$

The three vertexes of the triangular facet belong to this triangular facet:

$${EP}_{g}\in {EF}_{h}, {BP}_{i}\in {BF}_{j}$$

### The generation of distance vectors

The vector ($${V2}_{p},p=1\dots k)$$ is via point ($${EP}_{g})$$ and perpendicular to the triangular facet $${BF}_{j}$$. $$k$$ is the total number of vectors. The vector ($${V1}_{q},q=1\dots t)$$ is via point ($${BP}_{i})$$ and perpendicular to the triangular facet $${BF}_{j}$$. $$t$$ is the total number of vectors. The $${V1}_{q}$$ is defined as the distance vector and the absolute value of $${V1}_{q}$$ is $${S}_{q}$$. The segment which represents $${S}_{q}$$ is called the calculated segment:

$${EP}_{g}\in {V2}_{p}, {V2}_{p}\perp {BF}_{j}$$

$$(g=1\dots a, p=1\dots k,j=1\dots d,k<a,k<d)$$

$${BP}_{i}\in {V1}_{q}, {V1}_{q}\perp {EF}_{h}$$

$$\left(i=1\dots c, q=1\dots t,h=1\dots b,t<c,t<b\right)$$

$${S}_{q}=|{V1}_{q}|$$

### The discrimination of interference set and gap set

In order to discriminate the validity of distance vectors, the gap set is defined as ${G}_{1}$, ${G}_{2}$, and the interference set is defined as ${I}_{1}$, ${I}_{2}.$

When the angle between ${V2}_{p}$ and ${BV}_{j}$ is more than 90°,

$${EP}_{g}\in {G}_{2}, {BF}_{j}\in {G}_{2}$$

When the angle between ${V2}_{p}$ and ${BV}_{j}$ is less than 90°,

$${EP}_{g}\in {I}_{2}, {BF}_{j}\in {I}_{2}$$

The number of ${V1}_{q}$ is $m$, and the angle between ${V1}_{q}$ and ${EV}_{h}$ is ${\theta }_{n}(n=1\dots m)$.

When $$m=1$$ and $$\theta >90^\circ$$,

$${BP}_{i}\in {G}_{1}, {EF}_{h}\in {G}_{1}$$

When $$m=1$$ and $$\theta <90^\circ$$,

$${BP}_{i}\in {I}_{1}, {EF}_{h}\in {I}_{1}$$

When $m>1$ and all $\theta >90^\circ$,

$${BP}_{i}\in {G}_{1}, {EF}_{h}\in {G}_{1}$$

When $m>1$ and not all $\theta >90^\circ$,

$${BP}_{i}\in {I}_{1}, {EF}_{h}\in {I}_{1}$$

### The discrimination of validity

When all criteria listed below are satisfied, the overall gap set ($G$) is the ${G}_{1}$ plus the ${G}_{2}$. And the overall interference set ($I$) is the ${I}_{1}$ plus the ${I}_{2}$. The criteria are:

1. When ${EF}_{h}\in {V1}_{q}$, ${BP}_{i}\in {V1}_{q},{EP}_{g}\in {EF}_{h}\in {G}_{2},$ ${BP}_{i}\in {G}_{1},$ ${S}_{q}$ is saved.
  When ${EF}_{h}\in {V1}_{q}$, ${BP}_{i}\in {V1}_{q},{EP}_{g}\in {EF}_{h}\in {I}_{2},$ ${BP}_{i}\in {I}_{1},$ ${S}_{q}$ is saved.
2. $$Card\left({S}_{q}\bigcap EPM\right)=1$$
3. ${S}_{q}= \lambda {BV}_{j},$ $\lambda$ is the constant
4. the angle between ${S}_{q}$ and $EPM$ is more than 40°
5. $${BP}_{i}\notin {B}_{c}$$

### Statistical analysis and visualization

Based on the ${S}_{q}$ of the gap set ($G$) and interference set ($I$), the mean value, maximum value and standard deviation (SD) of $G$ and $I$, as well as the mean value, maximum value and SD of the whole set are calculated. All in all, there are eight statistical indexes, including the mean/max/standard deviation value of the gap set, the mean/max/standard deviation value of the interference set, and the mean/standard deviation value of all. For visualization, the segments of the interference set are shown in red and yellow, which appears that red represents a bigger gap and yellow represents a smaller gap. And the segments of the gap set are shown in blue and green, which indicates that blue represents a bigger interference and green represents a smaller interference.

**Figure 5:**
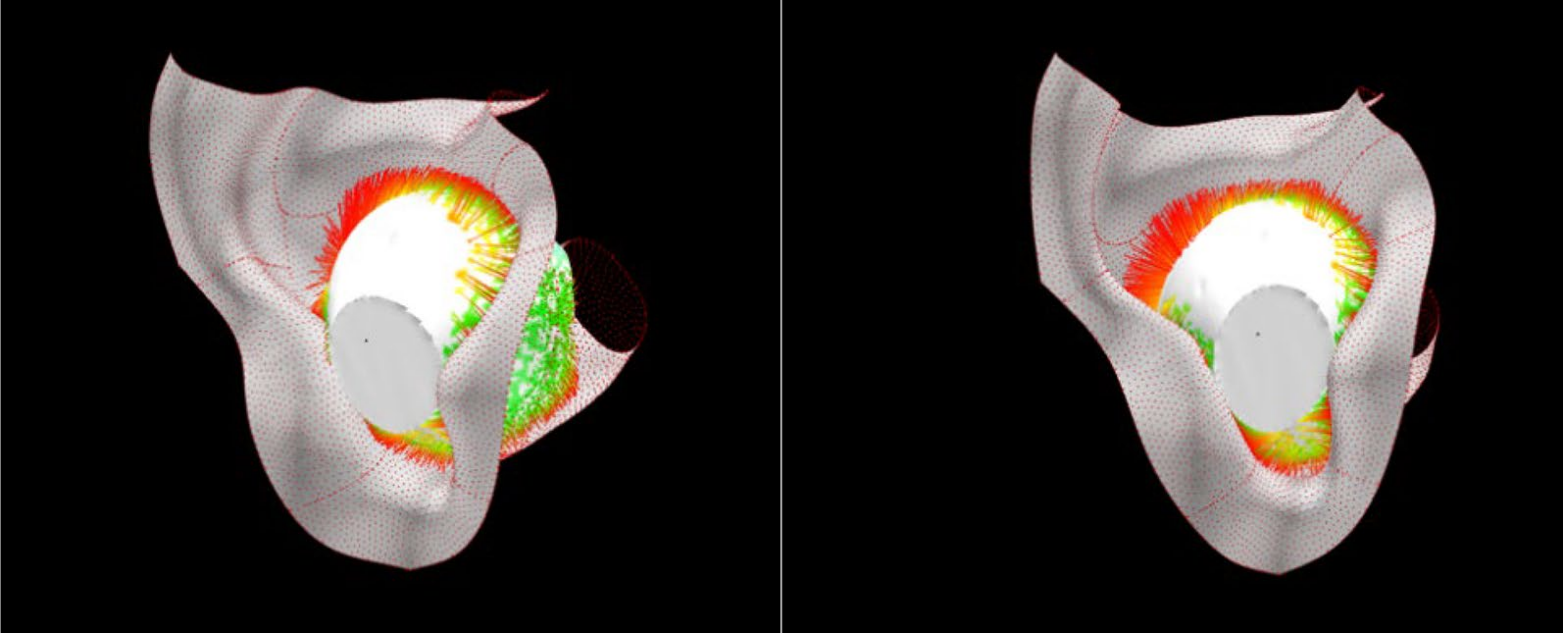
Calculation visual effect between earphone (with only body portion) and ear (with only concha part) in deviation analysis algorithm.

All programs were written in Node.js. Visually, each color line segment represents one distance value. The calculation visual effect between the earphone (with only the body portion) and ear (with only the concha and external auditory canal part) of the deviation analysis algorithm is illustrated in Fig. 5.

## Usability and validity verification of the deviation analysis algorithm

As mentioned in the pilot study, there is no industry or field standard of deviation analysis algorithm used for product fit analysis. In previous research, the deviation analysis functions of all commercial software were used for providing a tool for the product fit assessment. Hence, this algorithm should be verified to be used in fit assessment for earphone fit analysis.

To verify the usability and validity of the deviation analysis algorithm, an experiment on AirPods 2 (Apple Inc., California, length = 40.5 mm, width = 16.5 mm) was carried out. Previously, comfort perception for ear-related products was achieved through questionnaires^30^ or by reviewing online consumer content^31^. Thus, in this research, the Modified Borg scale was used for getting the comfort perception on AirPods 2. The deviation analysis algorithm was used for revealing the physical fit.

### Participants

34 young participants (16 males and 18 females) aged 21.182 ± 2.007 years old were recruited. This study was approved by the ethical review committee in the School of Design at Hunan University and conducted in Hunan University in accordance with the approval including all guidelines and regulations. All participants gave written and informed consent.

### Evaluation index

**Figure 6:**
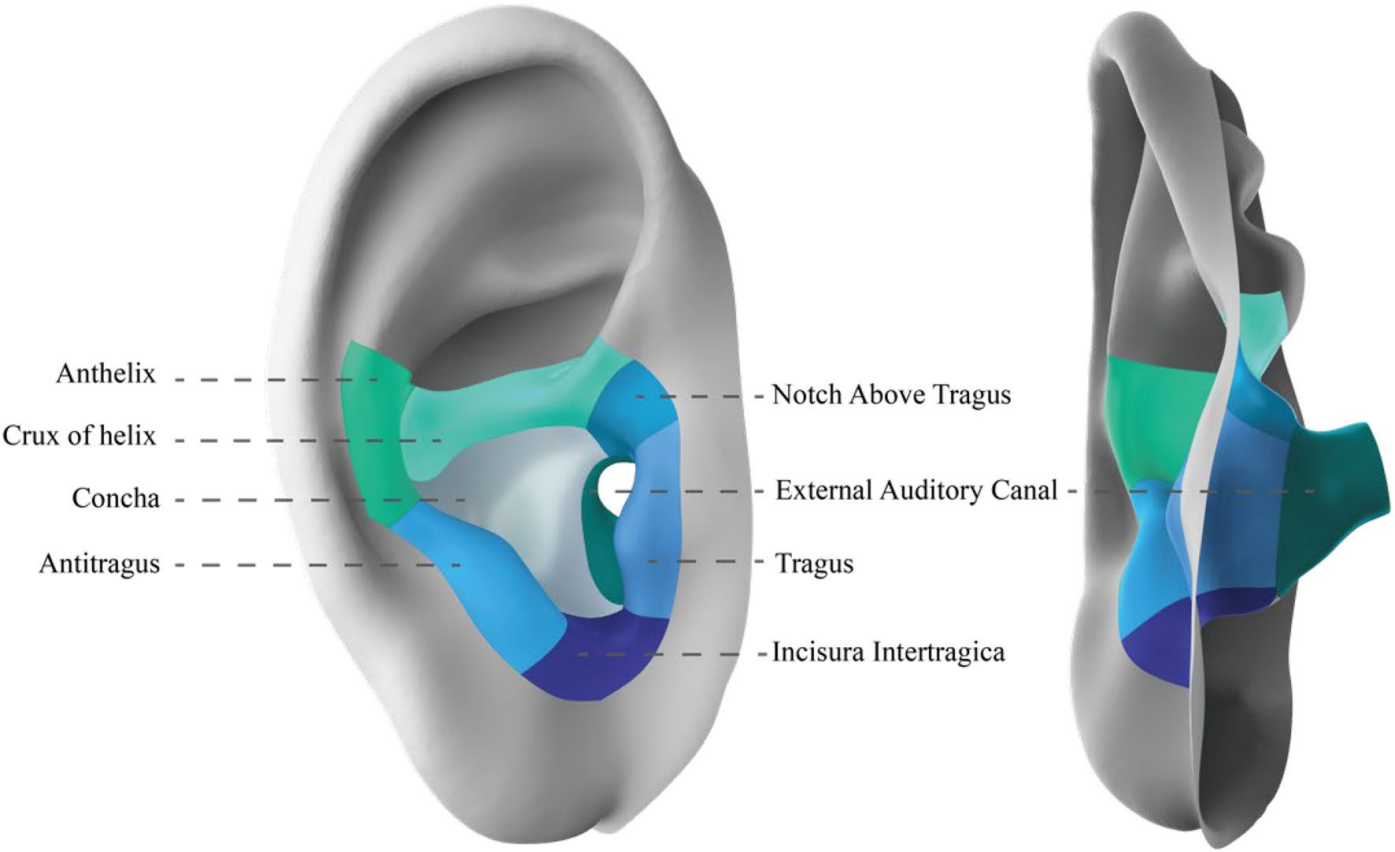
Eight anatomical ear regions.

Eight regions of the ear, including concha, anthelix, antitragus, crux of helix, notch above tragus, tragus, incisura intertragica and external auditory canal, were partitioned^32^, which is shown in Fig. 6. The regions are defined by anatomical landmarks and curvature of the ear according to ear anatomy^4,33^. Participants were asked to evaluate eight regions separately.

The measured variables in Borg CR-10 questionnaire^34,35^ were coded into quantitative data for analysis. Generally, there are four variables, including discomfort (0 = no discomfort, 10 = extremely strong (almost max) discomfort), sense of pressure (0 = no sense of pressure, 10 = extremely strong (almost max) sense of pressure), sense of stability (0 = no sense of instability, 10 = extremely strong (almost max) sense of instability), sense of volume (0 = no sense of volume, 10 = extremely strong (almost max) sense of volume) and the sense of pressure on eight regions of ear. Sense of pressure means the regional or holistic pressure sensation caused by earphones and sense of stability means that the earphone has a relatively stable relative position with the ear. In addition, the sense of volume represents the swelling and foreign body sensation caused by earphones.

### Experiment process

**Figure 7:**
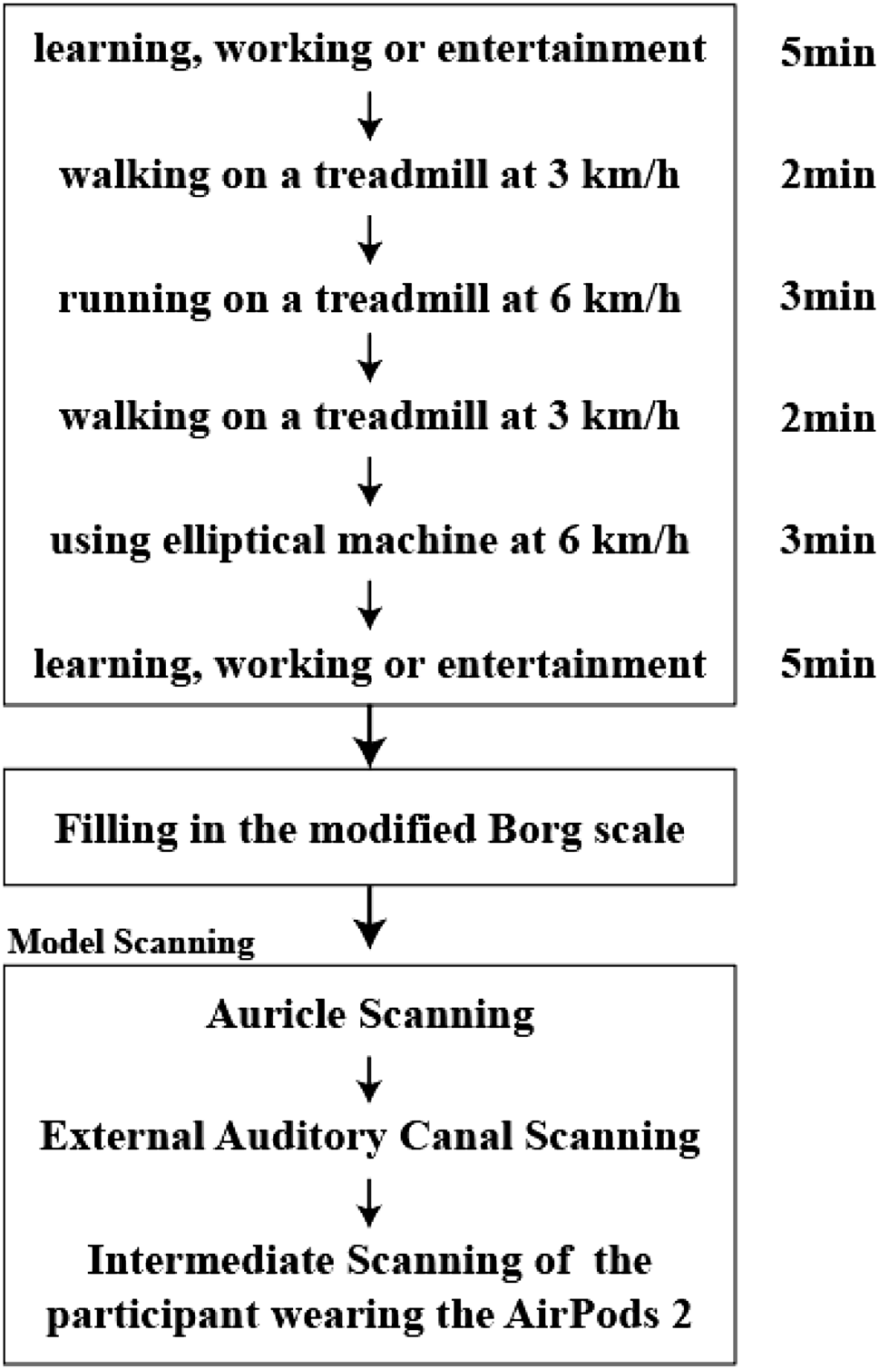
Evaluating process.

Participants were provided with AirPods 2. The duration of the experiment is about 20 min. After 20 min, participants were required to fill in the Borg CR-10 questionnaire. Straight after the questionnaire, model scanning was conducted immediately. There are three steps in model scanning, including auricle scanning, external auditory canal scanning, and intermediate scanning of the participant wearing the AirPods 2. Auricle scanning and intermediate scanning of participants wearing AirPods 2 were scanned by Artec Spider™ (Artec Group, Luxembourg) 3D scanner. In addition, external auditory canal scanning was conducted by 3Shape phoenix (3Shape Audio, Copenhagen) in-ear scanner for scanning the 3D shape of the concha and external auditory canal of the external ear. Averagely, the mean value of concha length is 29.67 for males and 29.34 for females. And the mean value of concha width is 19.89 for males and 19.43 for females^15^. The participants were asked to experience premeditated scenarios such as learning, working or entertainment, walking, running, and using the elliptical machine. It aims to imitate the actual scenarios when wearing an earphone. Similar tasks were assigned in previous studies^36–38^. The whole evaluating process is shown in Fig. 7.

### Data processing

**Figure 8:**
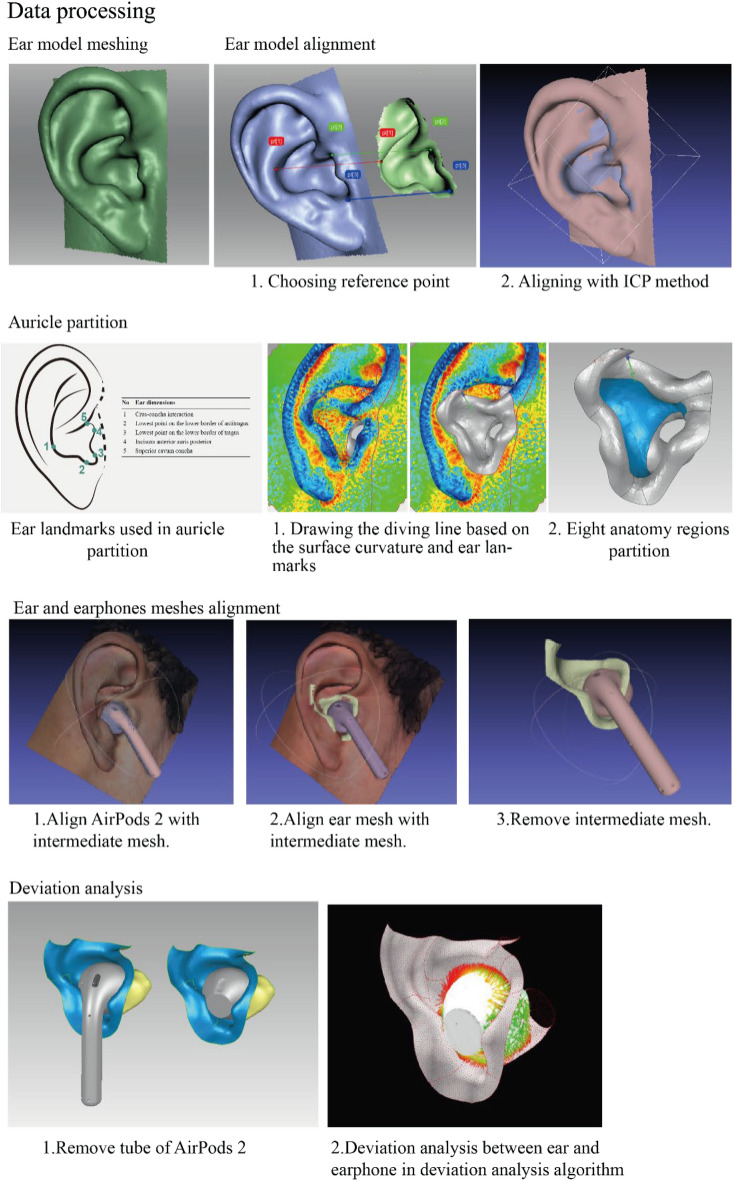
Data processing.

There are five main steps in data processing (Fig. 8), including ear model meshing, ear model alignment, auricle partition, ear and earphone meshes alignment, and deviation analysis.

*Deviation analysis* Before doing deviation analysis, the tube part of AirPods 2 was removed. The aligned body part of earphone meshes, overall ear meshes, and separate eight regions of ear mesh was imported into the deviation analysis algorithm. The mean value, maximum value, and standard deviation of gap sets and interference sets, as well as the mean and standard deviation of distances between ear and earphone, were calculated. The deviation analysis tool was used and the mean value, maximum value, of gap sets and interference sets, as well as the mean and standard deviation of all sets, were counted. Briefly, the deviation analyses were conducted nine times, including one overall analysis and eight analyses on separate regions of the ear with earphones.

After that, statistical analyses of deviation values calculated by the deviation analysis algorithm were conducted. (1) Descriptive statistics on deviation analysis values. (2) As the subjective evaluation score does not follow the normal distribution, the spearman correlation analyses were conducted between deviation analysis values and subjective discomfort scores.

### Result

First of all, the descriptive statistics (number, min, max, mean, and SD) were performed, as shown in Table 3. And spearman correlation analysis was conducted to understand the correlation between the values of the deviation analysis algorithm and the subjective discomfort score, shown in Table 4. It is shown that the mean value of all is statistically negatively correlated with comfort (r = −0.412, *p* < 0.05), which implies that comfortable experience is reported to be great when absolute values of gap and interference are small. Therefore, consistent morphological features between ears and earphones should be promoted. Besides, the maximum value of the gap is negatively correlated with comfort (r = −0.404, *p* < 0.05), which means that more interspace between ear and earphones brings evident discomfort feeling for people. In addition, the standard deviation of the gap is positively correlated with the overall sense of stability (r = −0.482, *p* < 0.01), which could be explained by the smaller morphological difference between ear and earphones, more stable earphones wear.

**Table 3:** The descriptive statistics of the result of deviation analysis algorithm.

Variable	N	Min. (mm)	Max. (mm)	Mean (mm)	SD (mm)
Maximum value of interference	215	− 4.179	− 0.202	− 1.812	0.861
Mean value of interference	215	− 2.545	− 0.114	− 0.819	0.423
Standard deviation of interference	215	0.047	0.955	0.440	0.211
Maximum value of gap	251	0.017	12.249	3.329	1.830
Mean value of gap	251	0.017	10.722	1.214	1.154
Standard deviation of gap	247	0.025	1.971	0.687	0.359
Mean value of all	258	− 2.476	10.722	0.344	1.437
Standard deviation of all	258	0.000	2.229	0.920	0.415

**Table 4:** Coefficient of spearman correlation between overall subjective evaluations and the results of deviation analysis algorithm.

Variable		Deviation analysis algorithm			
		Comfort	Sense of volume	Sense of stability	Sense of pressure
Maximum value of gap	Spearman Correlation	− 0.404*	− 0.116	0.134	− 0.115
	Sig.(2-tailed)	0.018	0.514	0.449	0.518
Standard deviation of gap	Spearman Correlation	− 0.062	0.064	0.482**	0.167
	Sig.(2-tailed)	0.728	0.718	0.004	0.344
Mean value of all	Spearman Correlation	− .412*	− 0.302	− 0.128	− 0.242
	Sig.(2-tailed)	0.015	0.083	0.471	0.167

**Correlation is significant at the 0.01 level.

*Correlation is significant at the 0.05 level.

The mean value of all eight different regions of ear meshes (including concha, anthelix, antitragus, notch above tragus, tragus, incisura intertragica, external auditory canal) was shown in Table 5. It is shown that the maximum value (5.685) of the mean value was shown in anthelix, which indicates that there is a huge gap between the earphone and the anthelix of the ear. Besides, it is also shown that the minimum value (-0.566) of the mean value was shown in the tragus, which indicates that there is an interference between the earphone and the tragus of the ear.

**Table 5:** The descriptive statistics on the deviation analysis result of deviation analysis algorithm.

Anatomy regions of ear	N	Min. (mm)	Max. (mm)	Mean (mm)	SD (mm)
Concha	34	− 1.133	0.918	0.362	0.453
Anthelix	8	2.410	10.722	5.685	2.922
Antitragus	34	− 2.476	1.611	− 0.076	1.019
Crux of helix	34	− 0.533	6.783	0.888	1.286
Notch above tragus	27	− 1.751	1.927	− 0.180	1.103
Tragus	34	− 1.858	1.894	− 0.566	0.736
Incisura intertragica	20	− 0.553	2.790	0.978	0.917
External auditory canal	33	− 1.235	1.103	0.074	0.623

**Correlation is significant at the 0.01 level.

*Correlation is significant at the 0.05 level.

Algorithm = Deviation analysis algorithm.

Spearman correlation analysis was conducted to understand the correlation between the values of the deviation analysis algorithm and subjective discomfort score for separate regions of the ear, shown in Table 6. For concha, crux of helix, and incisura intertragica of ear, comfort is negatively correlated with the mean value of concha (−0.378, *p* < 0.05) and incisura intertragica (−0.553, *p* < 0.05). The sense of volume is negatively correlated with the mean value of incisura intertragica (−0.609, *p* < 0.01). And the sense of stability is positively correlated with the mean value of crux of helix (0.402, *p* < 0.05). Besides, the overall sense of pressure is negatively correlated with the mean value of incisura intertragica (−0.603, *p* < 0.01).

**Table 6:** Coefficient of spearman correlation for mean value of three regions of ear.

Variable		Deviation analysis algorithm			
		Comfort	Sense of volume	Sense of stability	Sense of pressure
Mean value of Concha	Spearman Correlation	− 0.378*	− 0.311	-0.149	− 0.226
	Sig.(2-tailed)	0.027	0.073	0.402	0.199
Mean value of Crux of helix	Spearman Correlation	0.008	0.070	0.402*	0.206
	Sig.(2-tailed)	0.965	0.695	0.018	0.242
Mean value of Incisura Intertragica	Spearman Correlation	− 0.553*	− 0.609**	− 0.072	− 0.603**
	Sig.(2-tailed)	0.011	0.004	0.764	0.005

**Correlation is significant at the 0.01 level.

*Correlation is significant at the 0.05 level.

**Figure 9:**
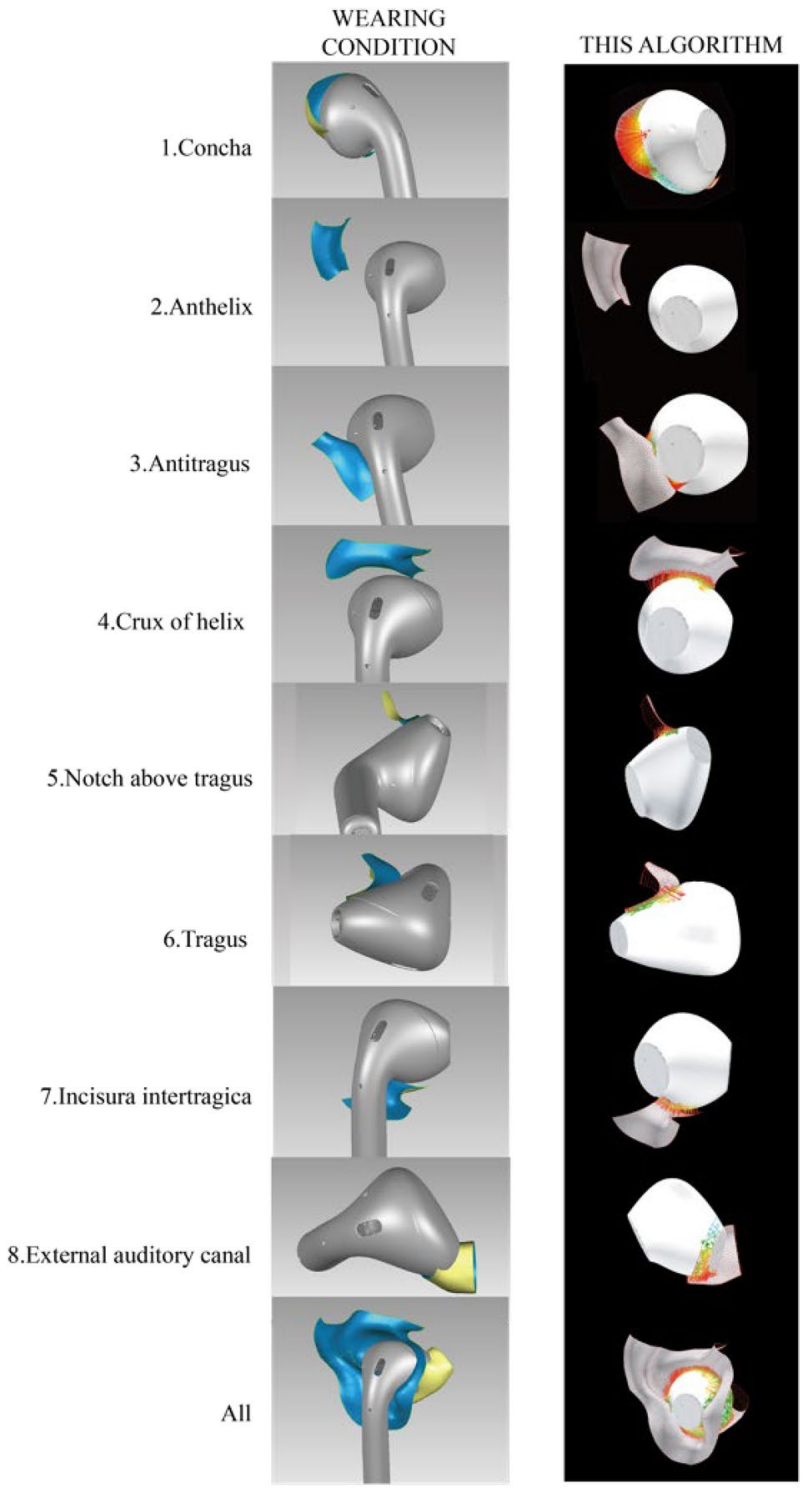
Screenshots of actual wearing condition (left), visual effect of deviation analysis by using deviation analysis algorithm (right).

Hence, the results in this study showed that the quantitative (deviation analysis value) and qualitative (participants’ comfort scores) data were related when comparing AirPods 2. This finding could demonstrate that the deviation analysis algorithm could be used for evaluating the fit of the earphone with the ear. in addition, the visual effect of deviation analysis between ear and earphone by using the deviation analysis algorithm is shown in Fig. 9.

## Discussion

In previous ergonomic design research, virtual fit and deviation analysis was frequently used for assessing product fit. In the research of Lee^11^, the comfort and fit of ear product design were predicted by analyzing the overlapping areas between ear-related products and ears by using the virtual fit method. In addition, In addition, the deviation analysis function in CATIA was used for analyzing the fit condition between the helmet and head^9^. However, seven different commercial software were used for doing the virtual fit and deviation analysis between earphone and ear. It turns out none of the existing deviation analysis functions of commercial software are appropriate for earphone fit analysis. Four common computational errors were summarized, which are shown in Fig. 2. Therefore, this research attempts to build a new deviation analysis algorithm that reflects the physical fit between ear and earphone more precisely. This algorithm could set the objects (ear model and earphone model) flexibly and effectively, discriminate the precise points and meshes that should be calculated, distinguish the interference and gap set, and provide eight indexes for evaluating earphone fit. And the anatomic characteristics of the external ear were taken into consideration in this algorithm. Therefore, this deviation analysis algorithm is the most optimal choice for earphone fit evaluation.

To verify the usability and validity of the deviation analysis algorithm, an experiment on AirPods 2 was conducted. In this experiment, the subjective comfort scores were rated by 34 participants. And the value of indexes of the deviation analysis algorithm was calculated. The correlation analysis between values of the deviation analysis algorithm and subjective comfort scores was conducted. And it found that there are statistically correlations between values of the deviation analysis algorithm and subjective discomfort score for both overall ear meshes and separate regions of the ear. When the absolute values of gap and interference are small and the maximum value of gap is comparatively small, the comfort scores are reported to be great. The SD of the gap in overall ear meshes and the mean value of crux of helix could be the indicators of the sense of stability. And the sense of pressure and volume could be implied by the mean value of incisura intertragica. As a whole, the deviation analysis algorithm can analyze the earphone fit objectively and the results calculated by this algorithm are statistically related to the participants’ feelings.

In the pilot study, the CATIA proved to be the best for earphone fit analysis statistically and visually. Therefore, the same well-aligned earphone and ear models were imported into the CATIA and deviation analysis algorithm for fit analysis. Overall ear mesh and eight regions of the external ear were calculated separately. Visually, the calculating effect of the deviation analysis algorithm is much better than that of CATIA.

**Figure 10:**
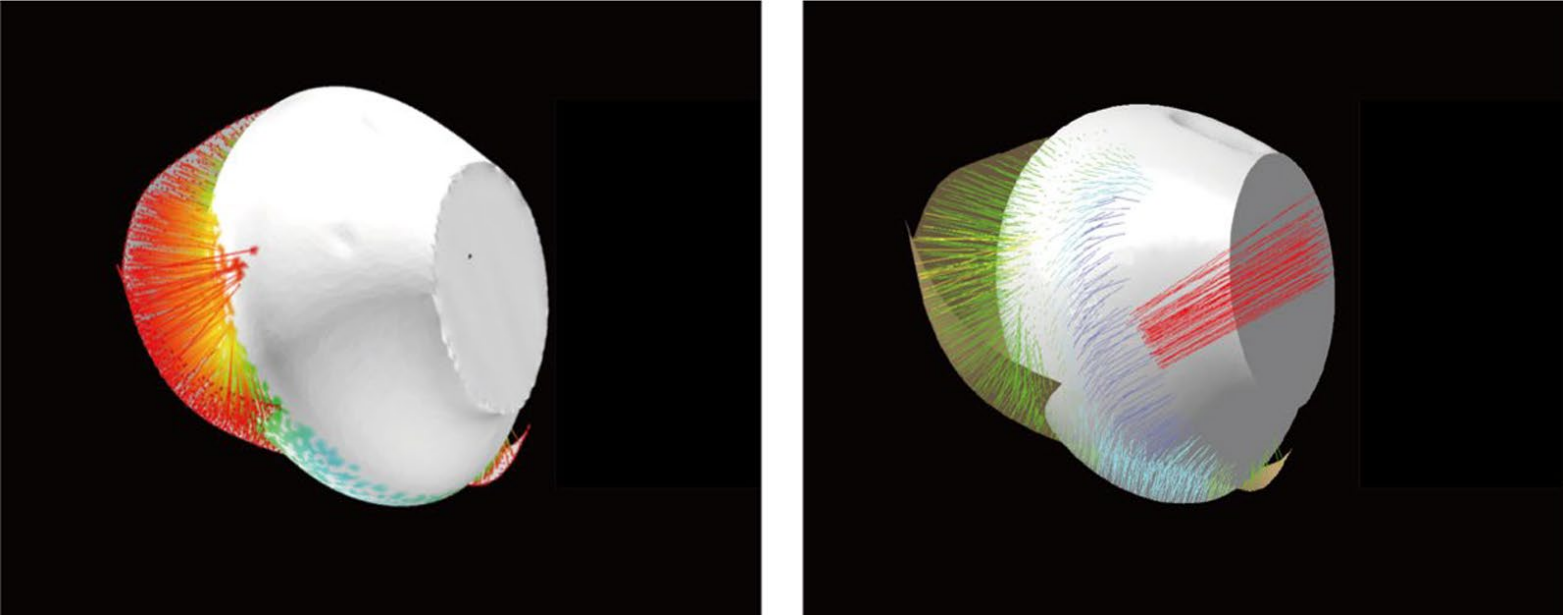
The visual effect between concha and earphone by deviation analysis tool of CATIA (right) and deviation analysis algorithm (left).

Firstly, the surfaces of both ear and earphone meshes are continuous. The deviation analysis results of adjacent point clouds of ear and earphone meshes should be close to each other. In the analysis results on the concha by deviation analysis tool in CATIA, it appears that some red lines are far away from other line segments, which is shown in Fig. 10 (right). This obvious error is revised in the deviation analysis algorithm, which is shown in Fig. 10 (left).

**Figure 11:**
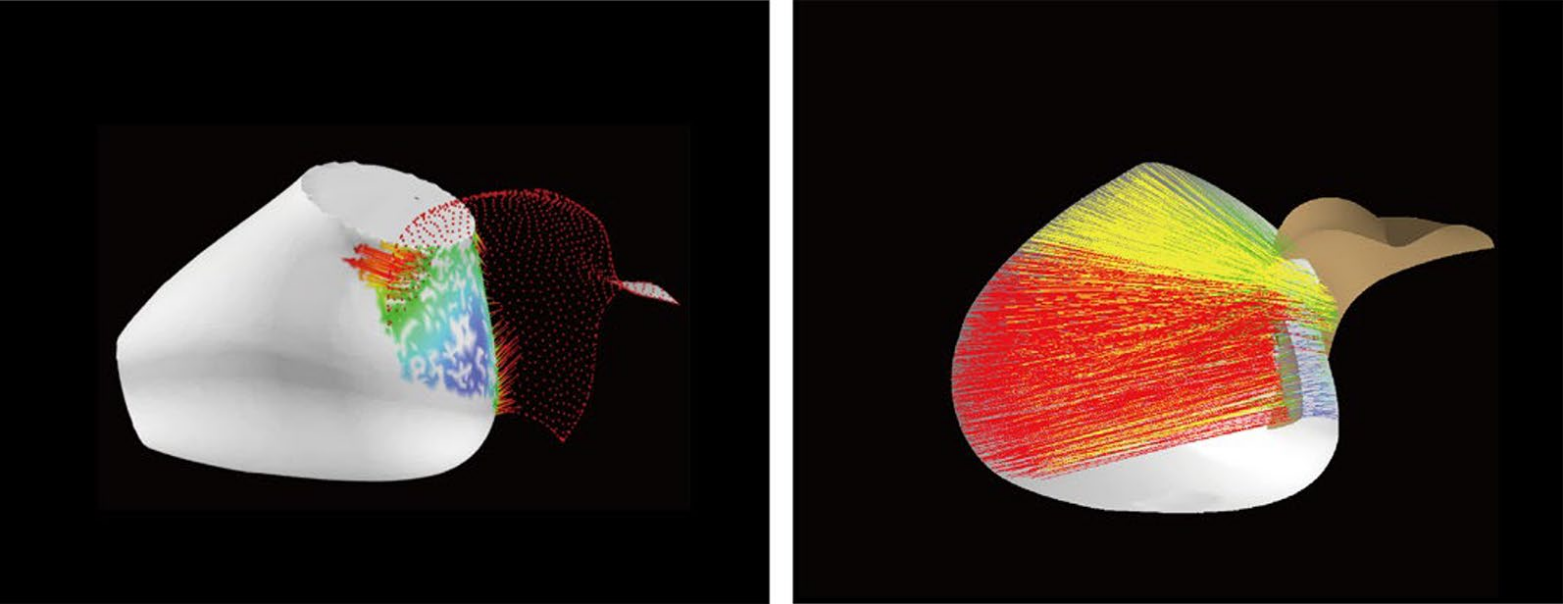
The visual effect between tragus and earphone by deviation analysis tool of CATIA (right) and deviation analysis algorithm (left).

Secondly, the points and triangle face of both ear and earphone meshes are separated into a set of gaps and a set of interferences in the deviation analysis algorithm. When both endpoints of the segment belong to the same set, the distance of this segment will be reserved for this set. On the contrary, in the CATIA, points and triangle faces are not classified. The surfaces of the ear sometimes were calculated with two surfaces of earphones when the surface of the ear embed into the earphone (Fig. 11 left). This mistake is amended in the deviation analysis algorithm (Fig. 11 right).

**Figure 12:**
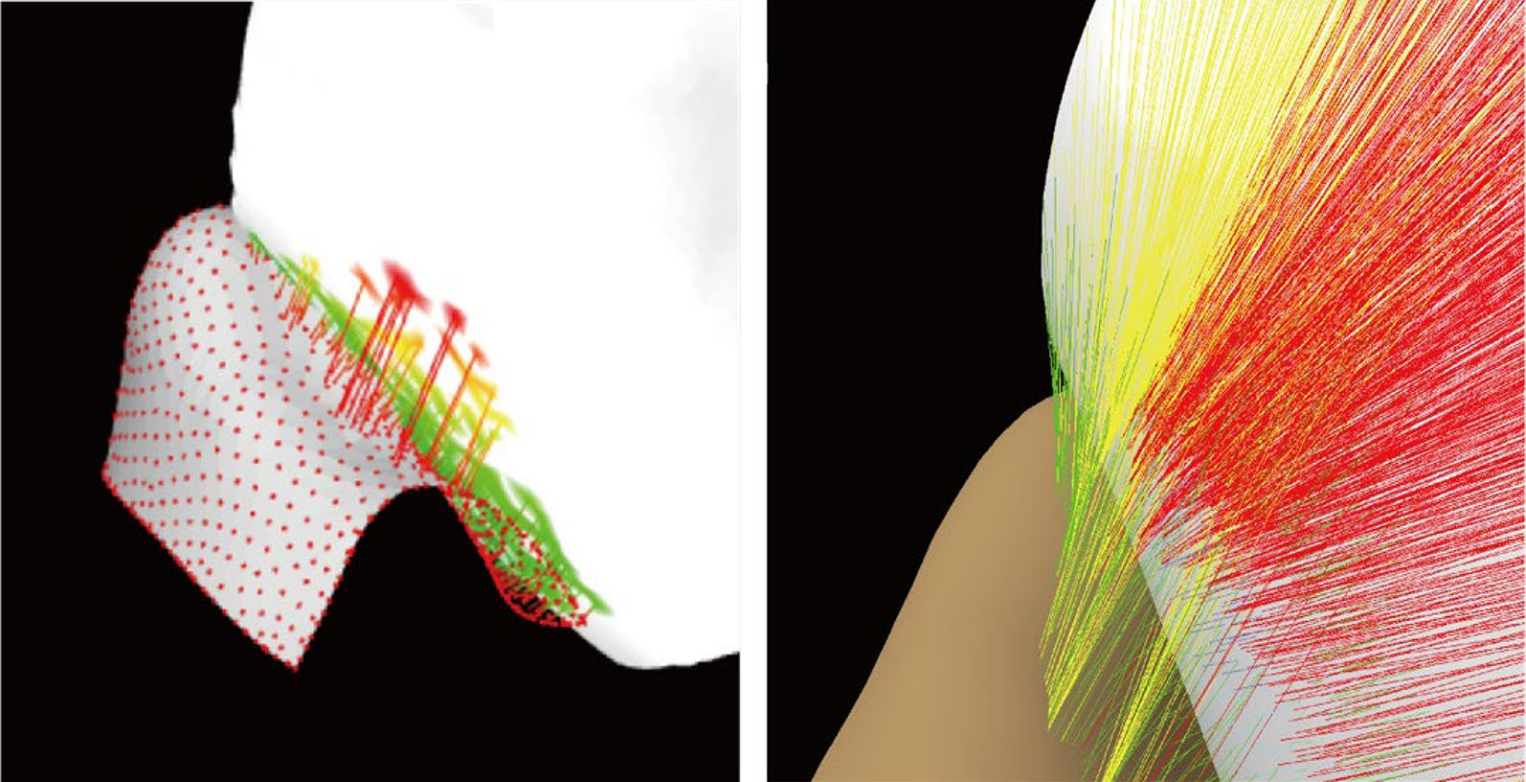
The visual effect between antitragus and earphone by deviation analysis tool of CATIA (right) and deviation analysis algorithm (left).

Furthermore, in both the deviation analysis algorithm and deviation analysis tool in CATIA, the angle between the line segment and ear meshes is 90°. The angle between the line segment and earphone meshes is set greater than 40° in the deviation analysis algorithm (Fig. 12 right). However, the angle between the line segment and earphone meshes has not been limited which could be any angle. Thus, some line segments are almost parallel with the surface of earphones in CATIA, which shows a much longer distance than it should be (Fig. 12 left). Generally speaking, the value of deviation analysis results between earphone and ear is better in the deviation analysis algorithm than in the deviation analysis tool in CATIA visually.

Certainly, the fit of the earphone could be improved according to the deviation analysis results. For the helmet, a Helmet Fit Index (HFI) was proposed, which is a novel method to investigate and compare the fitting accuracy of helmets based on deviation analysis between the helmet and the 3D model of head^9^. For revising mask boundaries, a new virtual fit approach was put forward, which defines horizontal distances between mask boundary and 3D face surfaces. It can assist in adjusting the oxygen mask boundary shape^39^. To improve the ergonomic design of earphones, the deviation analysis algorithm could predict subjective assessment of earphone fit partly. Through experience, statistical variables were calculated and correlated with perceived comfort ratings. For example, the overall comfort was negatively correlated with the maximum value of gap, which indicates that a smaller shape shows better comfort assessment. Besides, overall comfort was negatively correlated with the mean value of all as well, which implies that earphone fit well with ear shape revealing better comfort assessment. For different regions of ear, the mean value of concha is negatively correlated with overall comfort, which shows that the fitness of concha is vital for improving earphones. Consequently, the deviation analysis calculated by the deviation analysis algorithm between ear and earphones could be a great indicator for ergonomic design on earphones in improving comfort experience.

Besides, correlations between overall subjective discomfort evaluation and the deviation analysis variables are weak because similar subjective reviews on AirPods 2 are reported while diverse deviation analysis values are calculated. Generally, the complex biological structure of the ear (including fat, cartilage, skeleton), the character of earphone (material, weight, center of gravity of earphones, and shape of earphones), and user behavior^36^ should be considered as significant factors in affecting the comfort of wearing a wireless earphone. In this experiment, only the character of the earphone is considered which possibly resulted in a weakly significant result. Furthermore, the deviation analysis algorithm could be optimized and iterated with more data on earphones and ear meshes applied in the future. In the research of Yilmaz et al.^40^, the direction of deviated distance and form of distance were listed, which is set as shortest, along X, along Y, or along Z and set as the distance between a point and a triangle may consist of a point-to-plane, point-to-edge or point-to-vertex distance separately. In this research, the direction of deviation distance is set as the shortest distance between the points of earphones to the triangle faces of the ear. This deviation analysis algorithm could be improved based on more ear and earphones meshes virtual fit results and relative subjective discomfort score. More direction of deviation distance and the form of distance in deviation analysis could be studied in further research.

## Conclusion

This study promoted an accurate deviation analysis algorithm for the physical fit analysis of the earphone. There are five main procedures in this algorithm and it can work out eight indexes that represent the fit between earphone and ear. An experiment on AirPods 2 was conducted and it is shown that the values of the deviation analysis algorithm are statistically correlated with perceived comfort scores. Visually, this algorithm shows a better calculating effect than that of CATIA and the deviation analysis functions of other commercial software. The deviation analysis algorithm is certified to be the best choice for earphone fit analysis. Certainly, the earphone fit design could be improved with the help of this algorithm. And it is significant for ear-worn product design and development phases in the future.

## Data Availability

All data generated or analysed during this study are included in this published article.
